# Bodies bounce – deflection of bodies following first ground impact after falls from height

**DOI:** 10.1007/s00414-025-03484-4

**Published:** 2025-03-26

**Authors:** Lorenz Markus Bell, Markus Große Perdekamp, Ulrike Schmidt, Vanessa Thoma

**Affiliations:** Institute of Forensic Medicine, Medical Center, Faculty of Medicine, University of Freiburg, Albertstraße 9, 79104 Freiburg, Germany

**Keywords:** Case report fall from height, Second ground impact, Biomechanics of fall, Deflection, Rebound, Injury patterns after fall

## Abstract

Forensic examination of a scene where two bodies were found after a fall from a high-rise apartment building revealed a distinct tissue imprint resembling facial contours on the asphalt between the two bodies as well as a laceration on the back of the head and an abrasion on the forehead of one body. Tissue imprints located away from the positions of the bodies can indicate manipulation of the finding scene and injuries in opposing body regions at least two distinct blunt traumas. Initial assessment ruled out intermediate contacts with the building, pointing instead to a significant horizontal deflection of the bodies after the initial impact on the ground followed by a second impact. A review of the existing literature on falls from height was conducted, which provided limited information on the possibility of a body’s deflection after fall from height. Detailed investigations into the biomechanical relevance of sequences following that impact are rare. In contrast, surveillance video footage from the presented case shows the deflection of the corpses by as much as 1.5 m after initial impact, followed by a second impact. In combination with the autopsy results this provided a good explanation for the unusual forensic findings and unique biomechanical insights. It demonstrates that, depending on various factors like the impacted body region, ground structure and height of fall, a body can be significantly deflected from the initial impact region, resulting in a second impact and sometimes forensically misleading injury patterns.

## Introduction

The term “fall” is used in legal medicine to describe a downward movement of the body carried by high kinematic energy with abrupt, unrestrained impact on the ground [1]. Falls are a leading cause of unintentional injury death [2] and its subcategory “falls from height” represents a common method of suicide in many countries, especially in high-density urban areas with tall buildings [3]. Distinguishing between (extended) suicide, accident and homicide is necessary but complex [4], particularly when the fall is unobserved. The primary sources of information for reconstructing the manner and cause of death are the findings at the scene and during autopsy.

After falls from great height, in the vast majority of cases, injuries are already recognizable during the external examination [5], but in some cases the absence of externally visible injuries was described [1, 6, 7]. Authors report blunt injuries are usually confined to one surface plane of the body resulting from a “single impact on a flat surface” [5, 8]. Typical are open fractures of the soles of the feet and inguinal lacerations of skin and clothing [5]. The overall injuries like hematoma, skin abrasions, fractures and severe damage to internal organs often include several body regions [8, 9]. Fractures of the hyoid bone and the upper horn of the thyroid cartilage can occur, which is especially relevant in forensic context [5]. Several studies demonstrate how injury patterns after falls vary significantly with fall height. In falls from lower heights (less than 9–15 m), head injuries are most frequent, occurring in 79–84% of cases. In falls from greater heights multiple organ injuries and polytrauma become more prevalent (reported up to 92% at heights between 18 and 30 m). Türk et al. reported another increase of head injuries at heights above 25 m (in up to 90% of the cases). After falls from height head trauma is the dominant cause of death in lower falls, while polytrauma and trauma to the thorax become more prevalant at greater heights [5, 8, 9].

Reviewing the literature reveals important kinematic and kinetic aspects of falls from height, which we propose to divide into at least five key stages: (1) the initial situation, (2) the flight (phase), possible (3) intermediate (building) contacts, (4) the first ground impact and a possible (5) second ground impact. The different stages provide valuable insights for interpreting the nature of such falls, particularly in cases where severe injuries may obscure other critical details, where witnesses may be absent or unreliable and where not enough information is available for (numerical) simulation methods [10–13]:

### 1. Initial situation

Since the height of the fall determines the impact velocity and thus the kinematic energy of the impact, it represents the decisive parameter for the primary lethality and the injury pattern [1, 14]. A mortality rate of just over 50% is reported for a fall from the fourth floor (3.6 m floor height implied) onto solid ground. Falls from the fifth to sixth floor (3.6 m floor height implied) and higher onto solid ground are fatal in the vast majority of cases [14–16]. Suicides, on average, involve falls from greater heights than accidental falls which are more randomly distributed [5, 14, 17]. Türk et al. reported a mean height of about 10 m for accidental falls and of almost 23 m for suicides [5] while Berghaus stated that heights of 10 m and more are rather typical for suicides than for accidents [17]. Suicides tend to occur at the person’s home during the evening or at night, whereas accidents are more likely to happen at inadequately secured workplaces during daytime hours [5, 17]. If there is a barrier higher than the persons center of gravity, an accident is unlikely [18], yet falls over railings show a particularly mechanical complexity [19].

### 2. Flight (phase)

During the fall, there are both active and passive movements of the body, influenced by drag forces like air resistance and wind [10, 20]. However, according to Wach et al. active body movements have no direct influence on the track and rotation of the body during the fall [13].

### 3. Intermediate (building) contacts

During the fall, a body may come into an intermediate contact with building structures, such as canopies or crossbars. This results in both a loss of kinetic energy and additional injuries [13].

### 4. (First) ground impact

The first three phases and other factors such as ground structure and clothing, ultimately determine the resulting injury patterns. The kinetic energy is distributed more evenly during an impact with the entire front or back surface of the body than during a more localized impact with only one body region [9]. Notably, the point of impact after fall from heights is often at greater horizontal distance in suicides than in accidents, exceeding a distance of 1–2 m horizontally from the building. This phenomenon is often attributed to a forward jump in suicide cases, which was shown to have a relevant influence [17, 21, 22]. Wu Tsai et al. recently discussed a case where the influence of wind could explain a displacement of the body of up to 2 m after fall from a height of 30 m [20]. Numerical models show, that also without an active forward jump, a displacement of the body of up to 3 m is possible [10]. However, numerical models face challenges in the final phase of the simulations due to lacking accuracy in unloading phases when modeling rebound kinematics [10, 12].

In general only few studies consider the final phase of the fall discussing the possibility of deflection and **(5.) second ground impact** after first impact, none providing a reliably documented case on this topic [10, 13, 23]. While the first ground impact is typically prioritized when assessing trauma severity and causes of death, this approach may overlook processes that could explain discrete but forensically significant findings. For instance, injuries in opposing body regions might suggest at least two distinct traumas, necessitating consideration of more than one impact during the fall or the possibility of physical assault prior to the fall. Similarly, tissue traces found at unexpected locations around the body could be misinterpreted as manipulation of the body’s location at the scene when not evaluating the possibility of a horizontal deflection of a body after first ground impact. This report explores the relevance of biomechanical sequences following the first body impact on the ground. It provides a case where the deflection of two bodies after fall from height is fully documented by surveillance video footage, supplemented by autopsy findings and results of toxicological analyses.

## Case report

A resident who reported hearing a scream followed by two impacts found two deceased individuals in the courtyard of a high-rise residential building in an urban area. Until arrival of the forensic experts, no changes of the finding scene were made by medical or other involved personal.

### Scene description and circumstantial data

**Fig. 1:**
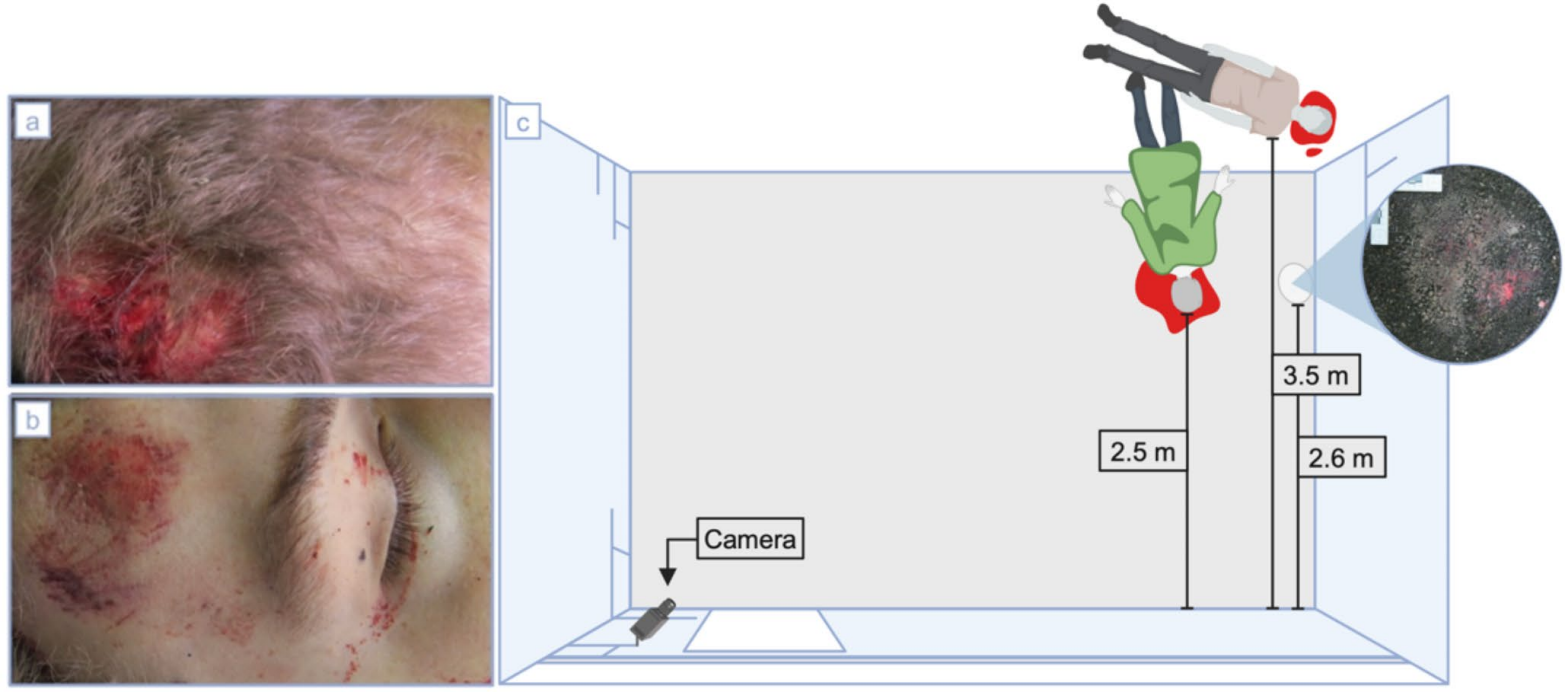
Finding site. On the left, injuries of the male with a laceration in the back of the head (**a**) and an abrasion on the forehead (**b**) are shown. Top view on the finding scene (**c**) shows the location of the bodies as well as the facial-like tissue imprint on the asphalt in between the two bodies. The bodies’ distances to the building were approximately 2.5 m (female) and 3.5 m (male). A surveillance camera had been installed close to a door to the staircase

The courtyard was located in the back of the twelve-story residential building with an H-shaped floor plan. All the walls bordering the courtyard are windowless and separate individual units. A common open staircase connects the units and is secured by a 1.2 m-high-railing. The courtyard is covered with asphalt. The bodies were found in supine (male) and prone (female) positions. The female body was discovered 2.5 m away from the staircase, while the male body was located 3.5 m from the staircase (Fig. 1). Subsequent identification showed they were a mother and her adult son, residents of an apartment on the fourth floor. The male had been diagnosed with depressive episodes.

### Scene traces, autopsy and toxicological data

At the eleventh floor a footprint on the railing and two parallel, rubber-like abrasion marks on the exterior of the building were found. Therefore, falls either from the eleventh floor, at a height of 31.3 m, or the fourth floor, at a height of 12.4 m, seemed plausible. However, the investigation revealed several unexplained elements. A tissue imprint was found on the asphalt between the two bodies with a distance of approximately 2.6 m away from the staircase and close to a sidewall of the building. Additionally, one body showed fresh injuries on opposing body regions, including the back of the head, forehead and chin (Fig. 1).

The autopsies of the two corpses were conducted three days later. The external examination of the female (156 cm, 63 kg) revealed tear-shaped skin wounds, hematomas and skin abrasions on the head, trunk and limbs. The female had suffered blunt craniocerebral trauma resulting in flattening and extensive skin abrasion of the face, contused lacerated wounds in the forehead region and in the frontoparietal region; also, a skull fracture accompanied by superficial brain tissue damage was present. The chest showed findings of a massive blunt trauma with bilateral serial fractures of the ribs, fractures of the spine and both shoulder blades. There was also a bilateral hemopneumothorax (670 ml blood in total), a rupture of the heart, a partial transection of the aorta and contusions of the lungs. Lacerations of liver and spleen caused an insignificant intraabdominal blood loss. Both upper and lower limbs, the right-sided pelvic ring as well as the laryngeal framework and the hyoid bone were fractured. Abnormalities in the bone substance were not noticed.

The external findings of the male corpse (184 cm, 105 kg) included contused lacerated wounds at the back of the head as well as several skin abrasions and décollements of the trunk. The internal examination revealed blunt trauma of the head with skull fractures accompanied by brain tissue damage as well as fractures of the cervical, thoracic and lumbar spine. The chest showed bilateral serial fractures of the ribs, lacerations of the lungs and diaphragm resulting in displacement of abdominal organs into the left thoracic cavity, partial and additional complete transections of the aorta, rupture of the heart and hematothorax (1 l of blood in total). In the abdomen, lacerations of the liver and the stomach, ruptures of the spleen and the kidneys were found. He also suffered a fracture in his left lower leg close to the knee. The bone substance was described as appropriate to his age.

The autopsy results were consistent with polytrauma as a consequence of a fall from great height. According to the extensive toxicological analyses, there was no evidence of drugs, drug agents or organic toxins.

### Video documentation and biomechanical analysis

**Fig. 2:**
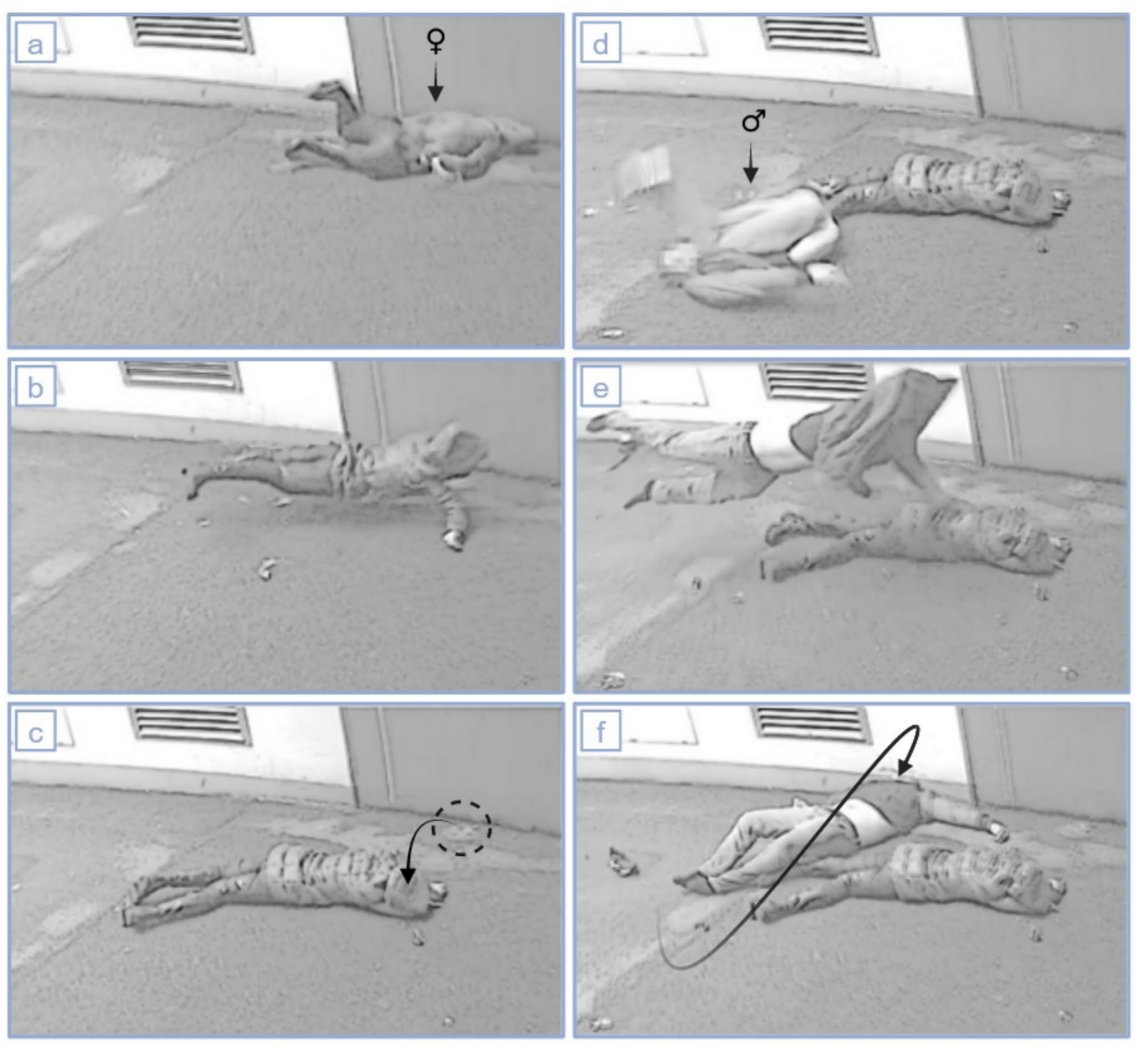
Surveillance video footage (screenshots of the video recordings were digitally edited to preserve anonymity). Shown are the female (**a**–**c**) and male person (**d**–**f**) at the moment of first ground impact (**a**, **d**), during the deflection sideways (**b**, **e**) and at the moment of second impact (**c**, **f**). The male body was deflected over a distance of slightly more than 1.5 m (marked by an arrow in image **f**). The deflection of the female body (marked by an arrow in image **c**) is small in comparison but left a visible tissue imprint on the ground (marked by a circle in image **c**)

Valuable evidence was made available through the presence of video surveillance. A camera positioned on the building’s exterior documented the events in question, allowing for an analysis of the surveillance footage (Fig. 2). The recordings revealed distinct patterns of impact for both persons on the flat, non-deformable ground surface. The female body struck with her entire front, leaving the tissue imprint of the head on the location of first ground impact and displaying a slight deflection of the body towards the camera afterwards. Approximately 1.5 s later, the male’s body landed on its upper back, exhibiting greater deformation of the ribcage, with legs bent to the maximum in front of the torso. From this point, the body underwent deflection and rotation along the sagittal plane, ultimately impacting once again, with an overstretched head and forehead, against the side wall. Overall, both bodies experienced significant horizontal deflection after their first ground impact, resulting in a second impact. Both bodies also experienced a vertical rebound, a reliable height estimation could not be determined. The male body, in particular, underwent a following rotation exceeding 180 degrees and a horizontal deflection by almost 1.5 m (when measuring the first ground impact point of the head compared to its final position). Notably, no visible traces from his first ground impact were observed at the finding site.

## Discussion

Final assessment of the actual fall height (fourth or eleventh floor) and of the falls‘ nature (accident, homicide or suicide) was not possible. The literature is still too ambiguous here, partly due to the high complexity of the topic. Overall, an accident seems unlikely in both cases, as in both cases the person’s center of gravity was below the 1.2-m-high railing. There were no indications of the involvement of a third party. In context with the psychiatric history of the male an (extended) suicide seemed plausible. Still, the unusual findings with the laceration on the back of the head and facial abrasions in the forehead of the male body as well as a facial-like tissue imprint on the ground in between the bodies necessitated further explanation. In the presented case, an intermediate impact on the building seemed unlikely due to its structural and geometrical features, such as the absence of balconies or other protrusions.

The surveillance footage showing a horizontal deflection and axial rotation of the male body after first ground impact provided a plausible explanation for those unusual findings. While secondary impacts are well-documented in context of car-pedestrian collisions [24], There is limited literature discussing a deflection of bodies after first ground impact in falls from height. Lau et al. indicate that determining the anatomical site of primary impact is not a straight forward matter, suggesting the possibility of a second impact and its complex interpretation [25]. In context of a case report, Wach et al. discussed a body’s rebound in context of simulation analysis of falls, noting that a significant rebound is possible when the body is in vertical or bending position (feet- or head-first fall) at first ground impact. However, they also argue that the analysis of films in real-life falling accidents of living humans shows that the body as an entity does not rebound at impact with a horizontal, non-deformable surface [13]. In experiments with dummies (model 3DGM-JM 50–67 from Ito Seiki with a length of 166,7 cm and a weight of 60,6 kg), Fujiwara was able to show that in his experimental setup bodies were deflected up to 1.2 m horizontally after fall from a height of 10 m when first impacting with the head and back [26]. Remarkable in that context is a retrospective study on fatal falls from heights in Berlin from 1988 to 2004 which found that the body changed position in 4.3% of the cases analyzed due to “rebound effects” which were not further specified [23]. To our knowledge, this case report is the first scientific analysis of a video-documented deflection of bodies after falls from a height. It opens up new insights into the biomechanical relevance of sequences after the initial impact on solid ground, which may be relevant for the forensic analysis of comparable cases in the future.

In conclusion, we observed a significant horizontal deflection of up to 1.5 m between the first and second ground impact locations of two persons following a fall from height onto a flat, non-deformable surface. The magnitude of the deflection appears to be particularly dependent on the body region impacted; a first ground impact with the elastic rib cage tends to be accompanied by greater deflection. The first ground impact is not necessarily marked by traces at the point of impact. The second ground impact can lead to additional and potentially misleading findings both at the site of discovery and on the corpse itself. Since our findings are only based on a single case study, their applicability to other scenarios is limited and experimental validation of the underlying biomechanical processes is necessary. However, the absence of traces at the first impact site suggests that the sole frequency estimate of such body deflections, at 4.3% [23], is most likely an underestimation. The case also shows that the distinction between a jump or a fall based on the distance between the body and the building, which is discussed in the literature, might not only be influenced by an active forward jump but also by deflection of the body. In general, the case illustrates the necessity of considering the possibility of deflection following the first ground impact in the analysis of falls from height, especially if the injuries are not limited to one surface plane of the body.

## Data Availability

Data underlying this article will be shared on reasonable request to the corresponding author.
